# Molecular characterization of bat HBV and identification of HDV co-infection in Yunnan, China

**DOI:** 10.3389/fmicb.2026.1763204

**Published:** 2026-02-10

**Authors:** Yushan Kui, Jiale Wang, Yuting Ning, Yutong Hou, Lulu Deng, Binghui Wang, Wen Li, Xueshan Xia

**Affiliations:** 1 Faculty of Life Science and Technology, Kunming University of Science and Technology, Kunming, China; 2 Yunnan Provincial Key Laboratory of Public Health and Biosafety, School of Public Health, Kunming Medical University, Kunming, China; 3 The Second Affiliated Hospital of Kunming Medical University, Kunming, China

**Keywords:** bat, co-infection, hepatitis B virus, hepatitis D virus, phylogenetic analysis

## Abstract

**Background:**

Bats are natural reservoirs for *Orthohepadnaviruses*, but the diversity and evolution of bat hepatitis B viruses (BtHBVs) and their co-infection with bat hepatitis D virus (BtHDV) in China remain poorly understood.

**Methods:**

In a molecular survey of bats from Yunnan Province, we detected BtHBV in 36.86% (129/350) of the samples, showing a notably high prevalence in *Hipposideros pomona* (41.75%). BtHDV co-infection was observed in 1.14% of BtHBV-positive bats.

**Results:**

Genomic analysis of 18 BtHBV isolates revealed that all belong to the bat-specific *Orthohepadnavirus* Lineage V (BtHBV-7), yet exhibit measurable genetic divergence. We also characterized a highly divergent bat HDV strain, BtHDV-YNNJ49, which clusters within a distinct bat-associated deltavirus lineage but displays substantial genetic divergence from its closest relative, underscoring diversity within this lineage.

**Conclusions:**

Collectively, our findings suggest that bats in Yunnan, particularly *H. pomona*, may act as natural hosts for both BtHBV and BtHDV. The identification of this divergent BtHDV strain expands our understanding of the genetic diversity and evolution of deltaviruses.

## 1 Introduction

Hepatitis B virus (HBV) is a partially double-stranded DNA virus from the *Orthohepadnavirus* genus and a member of the Hepadnaviridae family. HBV remains a major threat to global public health, affecting hundreds of millions of people and leading to high rates of chronic liver disease, cirrhosis, HBV-related cancer, and death. As of 2025, approximately 254 million people worldwide have chronic hepatitis B infection (Yan et al., 2025). China has a significant burden of HBV infection. According to a recent estimate, approximately 75 million people in China are infected with HBV, accounting for nearly one-third of the 254 million HBV-infected individuals worldwide. This high prevalence was attributed to the use of contaminated injections and blood transfusions between 1970 and 1992. With the introduction of the hepatitis B vaccine, the use of immunoglobulins, the advancement of medical technology, and increased awareness of HBsAg, the prevalence of HBsAg has decreased significantly from 9.7% in the early 1990s to 5.86% in the general Chinese population. Mother-to-child transmission has also decreased significantly, with a transmission rate of 0.23% in China. Among children aged 1–4, the HBsAg seroprevalence is currently only 0.3%, approaching the WHO’s 2030 elimination targets (Yan et al., 2025).

The evolutionary origins of HBV remain poorly understood, and no definitive zoonotic reservoir has been established. Phylogenetic studies suggest that human HBV infections have existed for approximately 15,000 years. In contrast, studies of endogenous avian hepadnavirus elements integrated into host genomes suggest a far deeper evolutionary history, extending back at least 40 million years (Locarnini et al., 2013). Hepadnaviruses infect a variety of mammals, including humans and certain primates (chimpanzees, gorillas, and gibbons) (Bonvicino et al., 2014; Lanford et al., 1998; Norder et al., 1996), several rodent species (including marmots, ground squirrels, and Arctic ground squirrels in the Americas) (Cohen et al., 1988; Seeger et al., 1984; Testut et al., 1996), and bats, which have been confirmed as natural hosts for numerous zoonotic pathogens (Lei et al., 2019; N’Dilimabaka, 2024; Nie et al., 2018). Although most animal hepadnaviruses exhibit strong host restriction, they serve as valuable models for understanding viruses’ origins and evolutionary dynamics. Notably, some animal HBV strains share a high genetic similarity with human HBV, suggesting that cross-species transmission and subsequent adaptation may have played a role in the emergence of human HBV infection. This evidence supports the hypothesis that human HBV may have originated from an ancestral zoonotic transmission (Norder et al., 1996).

Bat hepadnaviruses display notably greater genetic diversity than those found in other hosts. Multiple strains can coexist in a single bat species, and a single viral lineage may be distributed across diverse bat species, reflecting a long evolutionary history. Hepadnaviruses identified in New World bats share antigenic relatedness with HBV and can infect human liver cells (Drexler et al., 2013). Together with genetically diverse hepadnaviruses from New World rodents and non-human primates, these viruses exhibit a phylogenetic pattern where New World lineages often occupy basal positions. Rather than directly indicating a geographic origin, this pattern is more parsimoniously explained by the long-term preservation of early viral diversity under geographic isolation – a view supported by recent metagenomic studies revealing substantial hepadnavirus diversity in the New World (Ben et al., 2025). This pattern is compatible with the hypothesis that long-term geographic isolation may have preserved ancient viral diversity in the New World. Multiple host-switching events between bats and primates suggest that bats may be the source of the ancestral hepadnavirus in primates (Rasche et al., 2016). The frequent contact between bats and humans warrants continued vigilance and highlights the need for sustained virological surveillance.

Hepatitis D virus is a single-stranded circular RNA virus that lacks an independent envelope. It must rely on the HBsAg protein encoded by HBV to assemble into complete viral particles to achieve effective replication and transmission (Pan et al., 2023). Co-infection with both viruses often leads to more severe liver pathology and significantly increases the risk of severe hepatitis and hepatocellular carcinoma. Historically, this co-infection pattern has been considered exclusive to humans. However, recent studies have identified HDV-like viruses in various wildlife species. Their extensive genetic diversity suggests that the evolutionary history of HDV may be more complex than previously recognized.

We conducted a molecular epidemiological survey of BtHBV and BtHDV in bats from Yunnan Province, a key biogeographic region in China, to elucidate the genetic diversity, evolutionary patterns, and co-infection status of bat hepadnaviruses and deltaviruses.

## 2 Materials and methods

### 2.1 Sample collection

From 2021 to 2025, 350 bat specimens representing four species (spanning two genera and two families) were collected from three prefectures in Yunnan Province, China. The bats were euthanized by cervical dislocation after capture, and each specimen was individually sealed in a sterile bag. In the field, all samples were flash-frozen on dry ice, maintained during transport using refrigerated containers, and subsequently stored at −80 C°. During dissection under aseptic conditions, liver tissue–the primary replication site for hepadnaviruses–was collected, aliquoted, and preserved at ultra-low temperatures. All experimental procedures were conducted in strict accordance with the ethical guidelines for laboratory animal use and were reviewed and approved by the Animal Ethics Committee of Kunming Medical University (Approval no: kmmu20241037; Dated: March 11, 2024).

### 2.2 Viral nucleic acid extraction and taxonomic assignment

Approximately 25 mg of liver tissue was placed in a nuclease-free grinding tube and washed repeatedly with phosphate-buffered saline (PBS) to remove blood. Subsequently, 400 mL of PBS and an appropriate volume of grinding beads were added to each sample. The tissue was homogenized by bead-beating at 70 Hz for 60 s, and the process was repeated twice to ensure complete tissue lysis. Viral nucleic acids were extracted from the resulting homogenate using the Yifeixue Universal DNA/RNA Extraction Kit (Magnetic Bead-Based; Cat. No. YFXM0015), according to the manufacturer’s protocol. Bat species identification was confirmed by sequencing the mitochondrial cytochrome b (*Cytb*) gene (Irwin et al., 1991). The remaining nucleic acid extracts were stored at −80 C° for subsequent analysis.

### 2.3 Screening for BtHBV and BtHDV

Bat hepatitis B viruses screening employed nested polymerase chain reaction (PCR) primers (Supplementary Table 1) designed to target the HBV surface antigen gene (S gene; 299 bp in length) (Van Nguyen et al., 2018). BtHDV screening was performed using nested PCR targeting the hepatitis delta antigen (HDAg) gene, generating a 405 bp amplicon (Aftab et al., 2018) (Supplementary Table 1).

### 2.4 Sequencing of BtHBV and BtHDV

The complete genomes of bat HBVs (BtHBV) were amplified by nested PCR using specific primers (Supplementary Table 2). The full-length genomes of bat BtHDV were then sequenced using a high-throughput sequencing platform. All obtained BtHBV and BtHDV sequences discussed in this study have been deposited in the National Center for Biotechnology Information (NCBI) GenBank database, with the corresponding accession numbers provided in Supplementary Table 3.

### 2.5 Sequence alignment and phylogenetic tree analysis

**FIGURE 1:**
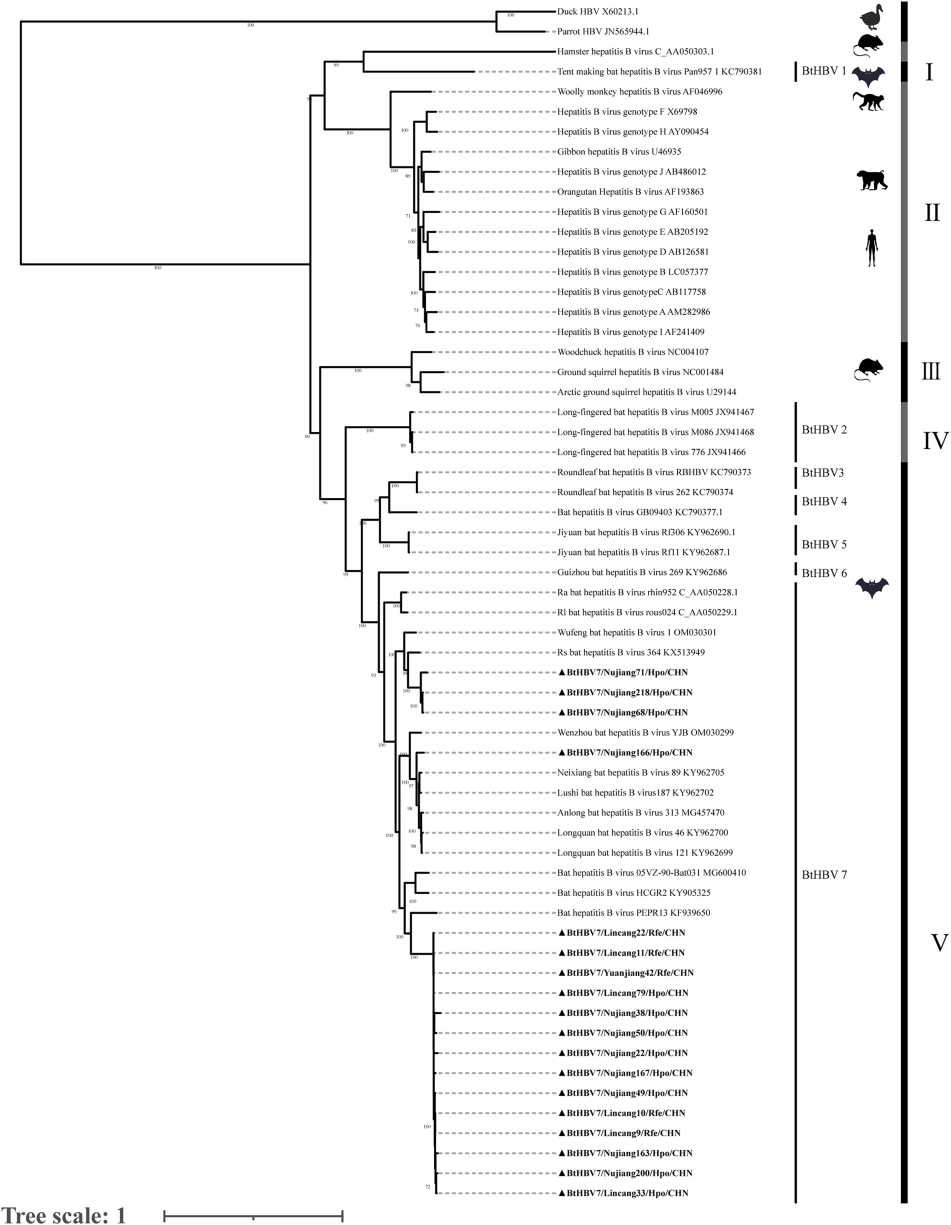
Phylogenetic analysis of the nucleotide sequences of the full-length genes of *Orthohepadnaviruses*. Phylogenetic trees were constructed using the maximum-likelihood method with the GTR+G model in IQ-TREE 3 software, with 1000 bootstrap replicates for statistical support. The host taxa and phylogenetic groups are shown in the mid-point-rooted tree.

Chromatogram quality control was conducted using the Chromas software, followed by sequence editing and contig assembly using the SeqMan software. After initial identification via BLAST on the NCBI platform, reference sequences were retrieved from GenBank and aligned using MAFFT (v7.520). Phylogenetic analysis was performed using IQ-TREE (v2.2.0), which implements the maximum likelihood method under the best-fit model selected by ModelFinder. All mammalian *Orthohepadnavirus* sequences (from humans, primates, bats, and rodents) were included as the ingroup for the *Orthohepadnavirus* (HBV) tree (Figure 1). The tree was rooted using two avihepadnavirus sequences (Duck HBV, X60213; Parrot HBV, JN565944) as the outgroup, following established methods (Ben et al., 2025; Lauber et al., 2017).

## 3 Results

### 3.1 Detection of BtHBV and BtHDV carriage rate

From 2021 to 2025, 350 bats representing four species, two genera, and two families were collected from designated regions in Yunnan (Table 1). The PCR assay detected BtHBV in 129 bats, corresponding to an overall prevalence of 36.86% (129/350). The infection rate varied by species, with 41.75% (124/297) observed in *Hipposideros pomona* (*H. pomona*) and 10% (5/50) in *Rhinolophus ferrumequinum* (*R. ferrumequinum*). HDV co-infection was identified in four bats, all of which were *H. pomona*, resulting in a BtHDV-positive rate of 1.14% (4/350) across the total sample and 1.35% (4/297) within this species (Table 1).

**TABLE 1:** Prevalence of BtHBV and BtHDV in different bat species.

Bat species	No. of samples	Location in Yunnan	PCR positive BtHBV	Rates of BtHBV (%)	PCR positive BtHDV	Rates of BtHDV (%)
*H. pomona*	297	Nujiang/Lincang	124	41.75	4	1.35
*R. ferrumequinum*	50	Lincang/Yuanjiang	5	10	0	0
R. *thomasi*	1	Nujiang	0	0	0	0
R. *affinis*	2	Nujiang	0	0	0	0

### 3.2 Phylogenetic analysis of the BtHBV

To further characterize the BtHBV strains identified in bats from Yunnan, 18 near-full-length genome sequences were successfully amplified. Sequence analysis showed that the genome size ranged from 3.2 to 3.4 kb. The genomic organization was consistent with that of *Orthohepadnaviruses*, containing the canonical open reading frames (ORFs) encoding the surface antigen (HBsAg, S region), core antigen (HBcAg, C region), DNA polymerase (P region), and X protein (X region).

Phylogenetic analysis based on full-length genome sequences clustered Hepatitis B virus into several major clades (Figure 1). Clades I, IV, and V contained bat viruses, Clade II comprised primate viruses, including all known human HBV genotypes, and Clade III consisted solely of rodent-associated viruses. In this study, all BtHBV strains identified in Yunnan Province fell within Clade V–a clade that harbors BtHBV genotypes 3, 4, 5, 6, and 7. Notably, these Yunnan strains clustered specifically within the BtHBV genotype 7 (BtHBV7) lineage, and could be further subdivided into several well-defined subclades. The topology of this BtHBV7clade was independently confirmed by phylogenetic analysis based on the viral polymerase (P) protein amino acid sequences (Supplementary Figure 1). Comparative phylogenetic analysis revealed that the Yunnan BtHBV7 strains shared the closest evolutionary affinity with other previously reported BtHBV7 strains in China, whereas they were only distantly related to other BtHBV genotypes (3–6) within Clade V.

Pairwise nucleotide identity analysis based on near-full-length genome sequences provided a quantitative assessment of these evolutionary relationships (Table 2). The three representative Yunnan strains (BtHBV7/Nujiang71/Hpo/CHN, BtHBV7/Nujiang166/Hpo/CHN, and BtHBV7/Lincang10/Rfe/CHN) shared a nucleotide identity of 81.84%–82.55%, whereas their identity with other Asian BtHBV strains within the same major clade (Clade V) ranged from 80.46% to 94.13%. In contrast, the identity between these Yunnan strains and representatives of other major clades was markedly lower: 54.31%–55.25% relative to Clade I (American bat viruses), 58.50%–60.69% relative to Clade II (primate viruses), and 34.23%–34.61% relative to Clade III (rodent viruses).

**TABLE 2:** Nucleotide homology analysis of tree BtHBV based on the full-length genome.

Virus strain	BtHBV7/ Nujiang166/ Hpo/CHN	BtHBV7/ Nujiang71/ Hpo/CHN	BtHBV7/ Lincang10/ Rfe/CHN	V-BtHBV_ anlong	V-BtHBVV_ wufeng	I-BtHBV	II-HBV_ human	II-HBV_ Orangutan	II-HBV_ monkey	III-HBV_ rodent	IV -BtHBV
BtHBV7/Nujiang166/Hpo/CHN	–	81.84	82.29	94.13	82.74	55.25	58.5	59.02	58.67	34.61	80.18
BtHBV7/Nujiang71/Hpo/CHN	81.84	–	82.55	82.41	85.97	54.31	59.51	59.26	60.69	34.41	80.69
BtHBV7/Lincang10/Rfe/CHN	82.29	82.55	–	83.43	80.46	55.08	59.17	59.52	60.31	34.23	81.99
V-BtHBV_anlong	94.13	82.41	83.43	–	83.91	55.22	59.24	59.71	59.5	34.64	81.08
V-BtHBVV_wufeng	82.74	85.97	80.46	83.91	–	55.26	59.4	60.44	60.44	34.59	80.6
I-BtHBV	55.25	54.31	55.08	55.22	55.26	–	53.12	53.72	53.95	31.08	54.44
II-HBV_human	58.5	59.51	59.17	59.24	59.4	53.12	–	88.39	76.22	33.99	59.35
II-HBV_Orangutan	59.02	59.26	59.52	59.71	60.44	53.72	88.39	–	78	33.06	59.77
II-HBV_monkey	58.67	60.69	60.31	59.5	60.44	53.95	76.22	78	–	33.95	60.26
III-HBV_rodent	34.61	34.41	34.23	34.64	34.59	31.08	33.99	33.06	33.95	–	33.79
IV-BtHBV	80.18	80.69	81.99	81.08	80.6	54.44	59.35	59.77	60.26	33.79	–

GenBank IDs for each virus are as follows: V-BtHBV_anlong (MG457470), V-BtHBV_wufeng (OM030301), I-BtHBV (KC790381), II-HBV_human (AM282986), II-HBV_Orangutan (AF193863), II-HBV_monkey (AF046996), III-HBV_rodent (NC001484), IV-BtHBV (KC790374).

### 3.3 Co-infection with BtHDV and BtHBV

Four cases of BtHDV co-infection were identified among the BtHBV-positive samples (Table 1). The initial 400 bp amplicons from these cases were identical. One representative strain (BtHDV-Nujiang49) was selected for full-genome sequencing and characterization.

**FIGURE 2:**
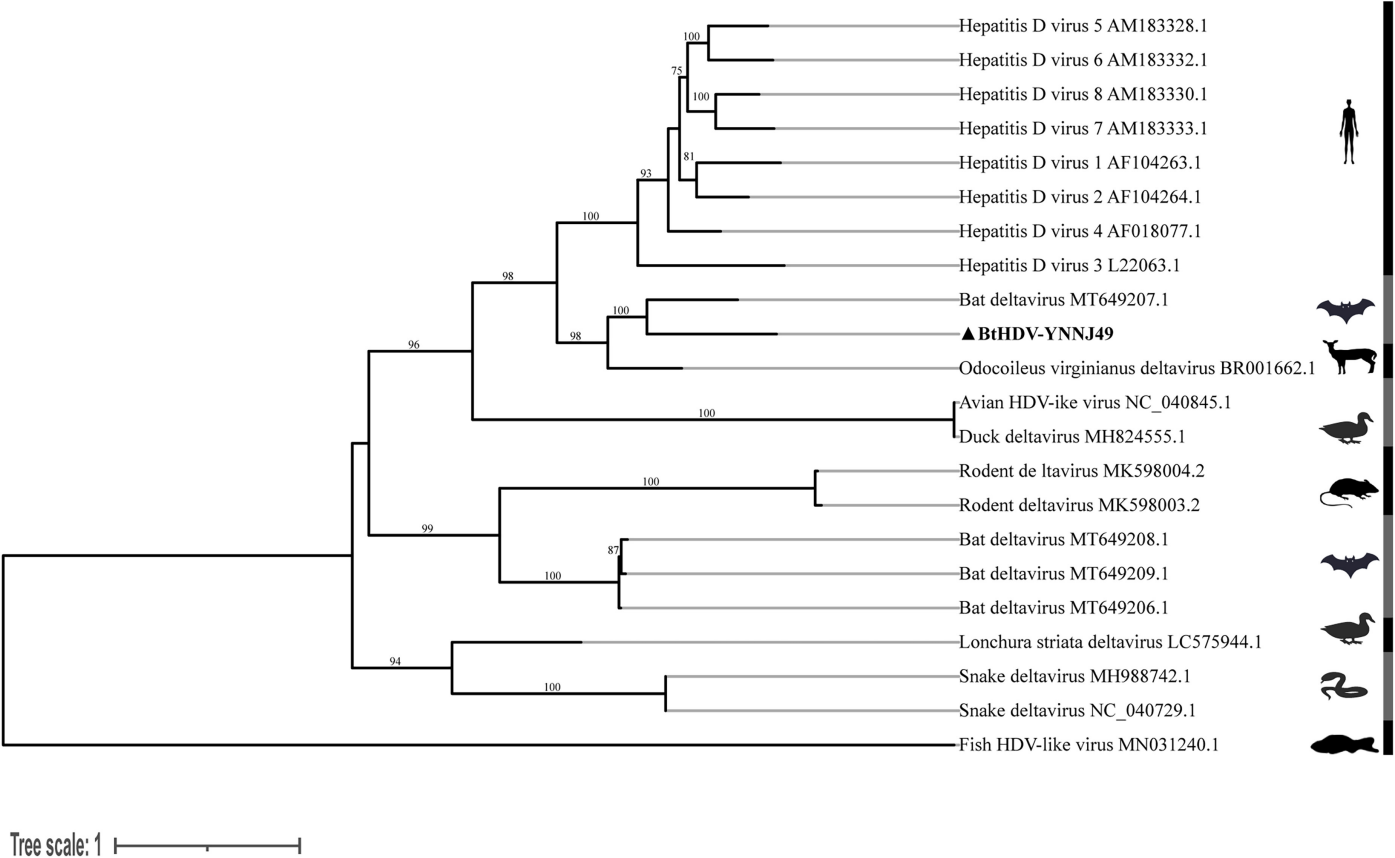
Phylogenetic analysis of the nucleotide sequences of the full-length deltavirus genes. Phylogenetic trees were constructed using the maximum-likelihood method with the GTR+G model in IQ-TREE 3 software, with 1000 bootstrap replicates for statistical support. The host taxa and phylogenetic groups are indicated. The trees were mid-point rooted for clarity.

Phylogenetic analysis based on the full-length genome showed that the human HDV sequences formed a strongly supported monophyletic clade. Bat-derived HDV strains clustered with those from ungulates and other mammals, whereas those from birds, rodents, and fish occupied more basal positions. The BtHDV-YNNJ49 strain formed a robust cluster with a previously reported HDV sequence from Peru (GenBank accession no. MT649207.1), constituting a distinct evolutionary lineage separate from human HDV (Figure 2).

Nucleotide and amino acid homology analyses (Table 3) revealed that BtHDV-YNNJ49 shared a maximum of 62.45% nucleotide identity and 68.21% amino acid identity with its closest known relative (Bat deltavirus AYA14). Its identities with another mammalian deltavirus (*Odocoileus virginianus* deltavirus) were 63.96% (nucleotide) and 73.33% (amino acid). In contrast, identities with HDV 3, other bat deltavirus (LMA6), rodent deltavirus, snake deltavirus, and Fish HDV-like virus were substantially lower, ranging from 30.76% to 55.69% (nucleotide) and 16.84%–54.42% (amino acid) than those with HDV 3.

**TABLE 3:** Nucleotide and amino acid homology analysis of BtHDV based on the full-length genome.

Virus strain	BtHDV-YNNJ49	Odocoileus virginianus deltavirus	Bat deltavirus AYA14	HDV 3	Bat deltavirus LMA6	Rodent deltavirus	Snake deltavirus	Fish HDV-like virus
BtHDV-YNNJ49	–	63.96/73.33	62.45/68.21	55.69/54.42	45.06/49.75	37.22/55.84	36.1/49.50	30.76/16.84
Odocoileus virginianus deltavirus	63.96/73.33	–	66.85/74.87	59.95/56.74	47.96/56.85	37.60/58.38	35.88/51.00	30.01/21.83
Bat deltavirus AYA14	62.45/68.21	66.85/74.87	–	55.62/53.95	47.3/56.85	36.85/55.84	35.58/52.00	30.27/19.29
HDV 3	55.69/54.42	59.95/56.74	55.62/53.95	–	43.65/48.85	34.76/50.23	36.87/45.00	29.80/15.21
Bat deltavirus LMA6	45.06/49.75	47.96/56.85	47.30/56.85	43.65/48.85	–	48.80/82.23	33.37/54.50	26.94/20.20
Rodent deltavirus	37.22/55.84	37.6/58.38	36.85/55.84	34.76/50.23	48.80/82.23	–	27.74/56.00	28.87/190.19
Snake deltavirus	36.10/49.50	35.88/51.00	35.58/52.00	36.87/45.00	33.37/54.50	27.74/56.00	–	23.60/16.00
Fish HDV-like virus	30.76/16.84	30.01/21.83	30.27/19.29	29.80/15.21	26.94/20.20	28.87/190.19	23.60/16.00	–

Values are shown as “nt/aa,” where “nt” denotes nucleotide homology (sequence similarity at the nucleotide level of full-length genes), whereas “aa” represents amino acid homology (sequence similarity of encoded proteins from corresponding nucleotide sequences). GenBank IDs for each virus are as follows: *Odocoileus virginianus* deltavirus (BR0016620.1), Bat deltavirus AYA14 (MT6492070.1), HDV 3 (L220630.1), Bat deltavirus LMA6 (MT6492090.1), Rodent deltavirus (MK598004.2), Snake deltavirus (NC_040729), Fish HDV-like virus (MN0312400.1).

## 4 Discussion

Hepatitis B viruses and HDV infections collectively represent a substantial global public health burden, accounting for a significant proportion of liver-related morbidity and mortality worldwide. HBV is the primary causative agent of chronic hepatitis, cirrhosis, and hepatocellular carcinoma, whereas HDV relies exclusively on HBV-derived surface antigens for its replication cycle and transmission. Clinical evidence firmly establishes that HBV/HDV co-infection is associated with accelerated progression to severe hepatic complications and significantly higher liver-related mortality rates than HBV monoinfection (Cho et al., 2023).

Our findings provide important empirical data from southwestern China in this context. The high prevalence of BtHBV (36.86%) detected in Yunnan bats, particularly exceeding 40% in *H. pomona*, strongly suggests that this species may be a key reservoir sustaining enzootic transmission.

Phylogenetic and pairwise identity analyses revealed that all Yunnan BtHBV strains belong to the bat-specific evolutionary Lineage V. Within this lineage, distinct subclades were formed by the characterized strains BtHBV7/Nujiang71/Hpo/CHN, BtHBV7/Nujiang166/Hpo/CHN, and BtHBV7/Lincang10/Rfe/CHN. A phylogenetic analysis based on the amino acid sequences of the HBV polymerase (P) protein consistently supported this clustering pattern (Supplementary Figure 1). The pairwise nucleotide identities among these representative strains ranged from 81.84% to 82.55%. Within Lineage V, the Yunnan strains, while sharing high nucleotide identity (80.46%–94.13%) with strains reported from other Chinese provinces, such as Guizhou and Zhejiang, formed distinct, well-supported subclades (Drexler et al., 2013; He et al., 2013; Nie et al., 2018; Yang et al., 2018). A critical finding from our expanded phylogenetic analysis was the distant relationship between these bat viruses and other mammalian *Orthohepadnaviruses*. The entire Lineage V clade showed substantially lower sequence identities compared with viruses of other mammalian orders: identities ranged from 58.50% to 60.69% with primate viruses (Lineage II) and approximately 34.00% with rodent viruses (Lineage III). A gradient of relatedness was evident when compared to other bat virus lineages: identities remained high with other Asian bat viruses (Lineage IV, 80.18%–81.99%), but were markedly lower with bat viruses from geographically distant regions, such as the Americas (Lineage I, 54.31%–55.25%) (Drexler et al., 2013).

However, these bat viruses are only distantly related to other mammalian *Orthohepadnaviruses*. For instance, the recently reported rodent strain (C_AA050303.1) (Ben et al., 2025), which is positioned on a deep, basal branch of the phylogenetic tree and clearly separate from the bat virus clusters identified in this study, also exhibits a distant evolutionary relationship with the latter. The substantially lower sequence identity between Lineage V and viruses of other mammalian orders underscores a long history of independent evolution within bats, aligning with the hypothesis that bats harbored ancestral hepadnaviruses prior to their diversification in other mammalian orders, a finding that is further highlighted by the intermediate identity with geographically isolated bat viruses (Lineage I) and underscores the combined roles of evolutionary time and geographic separation in shaping current viral diversity. The formation of phylogenetically distinct subclades among the Yunnan strains–despite their high overall identity with strains from other provinces–further supports a pattern of regionalized diversification, hinting at multiple independent local transmission chains rather than widespread cross-regional spread.

The current understanding of HDV remains limited and is predominantly based on human studies. As a satellite virus, the HDV depends on HBV co-infection to complete its replication cycle and establish infection in humans (Khalfi et al., 2023). Clinical evidence indicates that HBV/HDV co-infection significantly increases the risk of fulminant hepatitis and hepatocellular carcinoma compared to HBV monoinfection (Chiou et al., 2022; Gerber et al., 2021; Khalfi et al., 2023). At least three distinct BtHDVs have been documented in bat populations in previous studies. PCR-based screening of the corresponding samples failed to detect typical hepatoviruses in these bats. *In vitro* experiments have confirmed that hepatitis viral proteins can efficiently support HDV replication and virion assembly (Bergner et al., 2021; Pan et al., 2023). Although experimental HDV infection has been achieved in woodchucks carrying woodchuck hepatitis virus, natural co-circulation of HBV and HDV has not been documented in wildlife (Freitas et al., 2012; Negro et al., 1989). Consequently, HDV remains the only human RNA virus without a confirmed natural reservoir in other animal species. Establishing the auxiliary virus status of HDV-like agents in wildlife is essential for clarifying their evolutionary origins, host spectrum, and zoonotic potential, thereby advancing our understanding of their ecology and pathogenesis.

In this study, HDV RNA was detected in a subset of samples that were positive for BtHBV. Despite a low detection rate (1.14%), with all positive cases restricted to *H. pomona*, this finding strongly implicates this bat species as a potential reservoir of HDV. Nevertheless, whether HBV functions as a helper virus for HDV in wildlife remains unclear.

In addition, we also successfully obtained a near-complete genome sequence of a bat HDV strain (designated BtHDV-YNNJ49). Comprehensive phylogenetic and pairwise identity analyses revealed that BtHDV-YNNJ49 forms a robust, distinct clade with a previously reported bat HDV from Peru (Bergner et al., 2021) yet exhibits substantial sequence divergence (37.50% nucleotide difference, ∼31.80% amino acid difference). This pattern defines BtHDV-YNNJ49 as a highly divergent member within a unique bat-associated deltavirus lineage. The extent of genetic divergence highlights its distinctiveness and contributes to the known diversity of deltaviruses. Collectively, these findings provide new genomic data relevant to understanding the evolutionary history and ecological range of HDV-related viruses.

Taken together, this study and existing evidence underscore that bats are a crucial natural reservoir for hepadnaviruses. Their substantial viral diversity provides key insights into the long-term evolution of these viruses. Furthermore, our detection of a highly divergent bat HDV lineage reinforces that the ecology of deltaviruses in wildlife constitutes a critical dimension in assessing viral diversity and potential zoonotic pathways.

In summary, this study confirms that bats in Yunnan harbor a diverse community of *Orthohepadnaviruses* belonging to the bat-specific Lineage V (BtHBV 7) and documents the co-circulation of a highly divergent bat HDV lineage. These findings substantially expand the known genetic spectrum of both virus families in wildlife. They underscore that the evolutionary history of hepadnaviruses and deltaviruses in bats is complex and merits further investigation. Future research should focus on expanded surveillance to map the geographic and host distribution of these lineages, and on evolutionary analyses to elucidate their deep-time diversification and ecological dynamics. This work provides a foundation for such studies and reinforces the importance of sustained virological surveillance in wildlife for understanding viral diversity and emerging disease risks.

## Data Availability

The datasets presented in this study can be found in online repositories. The names of the repository/repositories and accession number(s) can be found in the article/Supplementary material.
