# Supplementary material for: Molecular characterization of bat HBV and identification of HDV co-infection in Yunnan, China
Source: Front Microbiol. 2026 Feb 10;17:1763204. doi: 10.3389/fmicb.2026.1763204 (PMC12929408; doi:10.3389/fmicb.2026.1763204)
Supplement: Supplementary file 1 [file Data_Sheet_1.docx]

Supplementary Material

**Table S1. Primers and PCR product lengths for BtHBV, BtHDV.**

| **Target** | **Primer** | **Sequence (5' - 3')** |
| --- | --- | --- |
| HBV | HBV_FW-266 | GTGGTGGAYTTCTCWCARTT |
|  | HBV_RW-763 | CCCCAAWACCANRTCATCCATA |
|  |  |  |
|  | HBV_FN-367 | GTCTCTGCGGACGAGATAACCTC |
|  | HBV_RN-687 | CTAGTAAAYTGAGCCARGAGAAA |
| HDV | HDV_F1 | GCCCAGGTCGGACCGCGAGGA |
|  | HDV_R1 | ACAAGGAGAGGCAGGATCACCGAC |
|  |  |  |
|  | HDV_F2 | GAGATGCCATGCCGACCCGAAGAG |
|  | HDV_R2 | GAAGGAAGGCCCTCGAGAACAAGA |

**Table S2. Primers for amplification of complete genome of HBV.**

|  | **Primer** | **Sequence (5' - 3')** |
| --- | --- | --- |
| 1 | HBVF1-26 | ARGACACTCATCCACATCTCAACATG |
|  | HBVR1-786 | GAACAAACAGAGGGCATAAAACGGACT |
|  | HBVR2-683 | TGAATTGAGCCAGGAGAAACGG |
| 2 | HBVF2-1762 | TAATGAATCAATGGGAGGAGACRGG |
|  | HBVF1-1570 | CTGTTCGTCGTCTCCCGC |
|  | HBVR2-2324 | GGAAGAGTTGAGAGAATGGGTGC |
|  | HBVR1-2439 | GAGTTCTTCTGCGACGCGGTG |
| 3 | HBVF3-2346 | TGCACCCATTCTCTCAACTCTTCC |
|  | HBVF4-2407 | CTACTAGGTCCCCCAGAAGACGC |
|  | HBVR4-3259 | GGTGGAGGAGGCAAAAGGT |
|  | HBVR3-3309 | TTGCTGGTARBGWDGGGGGGCG |
| 4 | HBVF1-450 | ATGCCTCATTTGCTTGTTGG |
|  | HBVF2-591 | TGTTGTACAAAACCTACGGGAG |
|  | HBVR2-1190 | AGTGGGGGGTTGCGTCAGC |
|  | HBVR1-1213 | GAGTTGATGATTAACCAGGCCC |
| 5 | HBVF5-2397 | GCTACTAGGTCCCCCAGAAGAC |
|  | HBVF6-2424 | CCTCTCCTCGCAGACGCAGA |
|  | HBVR6-3253 | GGTGGAGGAGGCAAAAGGT |
|  | HBVR5-3317 | TTGCTGGTARBGWDGGGGGGCG |
| 6 | HBVF1-683 | CCGWTTCTCYTGGCTCAATTC |
|  | HBVF2-763 | ATGGATGATGTGGTWYTGGG |
|  | HBVR1-1779 | TGCCTACAGCCTCCWRATACATATG |
|  | HBVR2-1587 | CACAGGTGAADCGAAGKRCACAC |

**Table S3. The GenBank accession numbers of the strains.**

| **No.** | **Strains name +** | **Gene GenBank accession numbers** |
| --- | --- | --- |
| 1 | BtHBV7/Nujiang22/Hpo/CHN | PP373707.1 |
| 2 | BtHBV7/Nujiang38/Hpo/CHN | PX590522 |
| 3 | BtHBV7/Nujiang49/Hpo/CHN | PX590523 |
| 4 | BtHBV7/Nujiang50/Hpo/CHN | PX590524 |
| 5 | BtHBV7/Nujiang68/Hpo/CHN | PX549096 |
| 6 | BtHBV7/Nujiang71/Hpo/CHN | PX590520 |
| 7 | BtHBV7/Nujiang163/Hpo/CHN | PX590525 |
| 8 | BtHBV7/Nujiang166/Hpo/CHN | PX590526 |
| 9 | BtHBV7/Nujiang167/Hpo/CHN | PX549095 |
| 10 | BtHBV7/Nujiang200/Hpo/CHN | PX590527 |
| 11 | BtHBV7/Nujiang218/Hpo/CHN | PX619212 |
| 12 | BtHBV7/Lincang9/Rfe/CHN | PX590519 |
| 13 | BtHBV7/Lincang10/Rfe/CHN | PX590528 |
| 14 | BtHBV7/Lincang11/Rfe/CHN | PX549093 |
| 15 | BtHBV7/Lincang22/Rfe/CHN | PX590521 |
| 16 | BtHBV7/Lincang33/Hpo/CHN | PX549092 |
| 17 | BtHBV7/Lincang79/Hpo/CHN | PX549090 |
| 18 | BtHBV7/Yuanjiang42/Rfe/CHN | PX549091 |
| 19 | BtHDV-YNNJ49 | PX619211 |

## Figures

**Supplementary Figure 1.** Phylogenetic analysis based on the amino acid sequences of the HBV polymerase Phylogenetic trees were constructed using the maximum-likelihood method with the GTR+G model in IQ-TREE 3 software, with 1000 bootstrap replicates for statistical support. The host taxa and phylogenetic groups are shown in the mid-point-rooted tree**.**
