# Supplementary figures and images for: Molecular characterization of bat HBV and identification of HDV co-infection in Yunnan, China
Source: Front Microbiol. 2026 Feb 10;17:1763204. doi: 10.3389/fmicb.2026.1763204 (PMC12929408; doi:10.3389/fmicb.2026.1763204)

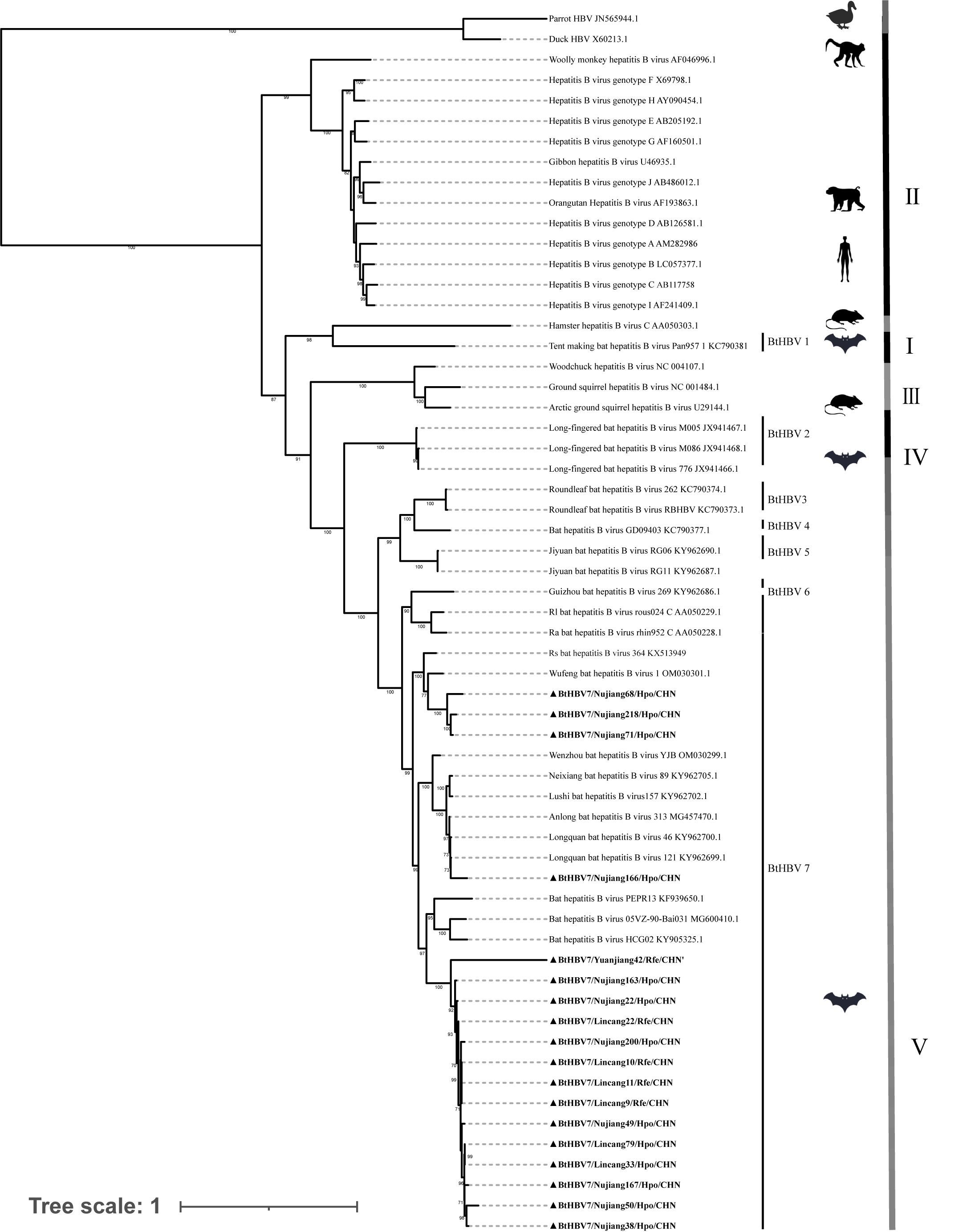

Supplement: Supplementary file 2 [file Image_1.tif]
